# Supplementary material for: High Incidence of Metastatic Infections in Panton-Valentine Leucocidin-Negative, Community-Acquired Methicillin-Resistant Staphylococcus aureus Bacteremia: An 11-Year Retrospective Study in Japan
Source: Antibiotics (Basel). 2023 Oct 6;12(10):1516. doi: 10.3390/antibiotics12101516 (PMC10604685; doi:10.3390/antibiotics12101516)
Supplement: Supplementary file 1 [file antibiotics-12-01516-s001.zip › antibiotics-2606021-supplementary.pdf]

Supplementary Material

Figure S1. Annual trends in the proportions (%) of methicillin-resistant *S. aureus (*MRSA) among all *S. aureus* isolates at Toyama University Hospital.

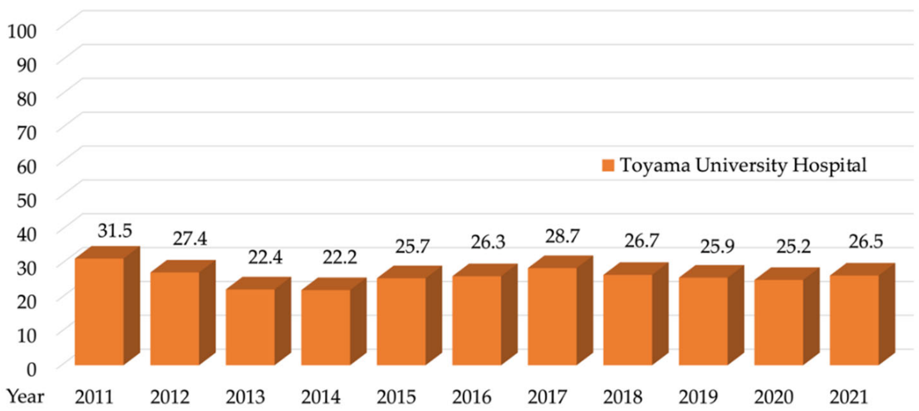

Table S1. Details of metastatic infections.

SCC*mec* type, n (%)	Details of metastatic infections	Number of cases
II	Vertebral osteomyelitis	1
	Knee arthritis and endophthalmitis	1
IV	Vertebral osteomyelitis / iliopsoas abscess	3
	Infective endocarditis	3
	Septic pulmonary embolism / lung abscesses	2
	Infected aortic aneurysm	1
	Endophthalmitis	1
	Subcutaneous abscess	1
V	Vertebral osteomyelitis and kidney abscess	1
VI	Infective endocarditis	1
VIII	Vertebral osteomyelitis	1
Non-typable	Infected aortic aneurysm and iliopsoas abscess	1
	Posterior pharyngeal abscesses	1

SCC*mec*, Staphylococcal cassette chromosome *mec*

Table S2. Relationships between the carriage of *cna* and *fnbB* and the development of metastatic infection in CA-MRSAB.

Virulence gene	Number of strains in CA-MRSA (n = 36), n (%)	With metastatic infection, n =12, n (%)	Without metastatic infection, n = 24, n (%)
cna (+)	20 (76.9)	7/20 (35.0)	13/20 (65.0)
cna (–)	16 (24.1)	5/16 (31.25)	11/16 (68.75)
fnbB (+)	18 (50.0)	6/18 (33.3)	12/18 (66.7)
fnbB (–)	18 (50.0)	6/18 (33.3)	12/18 (66.7)

CA-MRSAB, community-acquired methicillin-resistant *S. aureus* bacteremia

Table S3. Demographic and clinical characteristics of patients with bacteremia caused by CA-MRSA with and without *cna*.

Characteristics	*cna* (–), n = 12	*cna* (+), n = 15	*P* value
Age, yrs, median (IQR)	70.5 (54–84)	70 (59–76)	0.54
Sex, male, n (%)	7 (58.3)	12 (80.0)	0.40
BMI (kg/m^2), median (IQR)	21.4 (16.3–22.8)	20.0 (17.2–23.1)	0.92
Acquisition of infection, n (%)			
Hospital-onset	7 (58.3)	22 (61.1)	0.46
Source of infection, n (%)			
CRBSI	7 (58.3)	6 (40.0)	0.45
Lower respiratory tract	4 (33.3)	3 (20.0)	0.66
Surgical site	0 (0.0)	0 (0.0)	–
Infective endocarditis	0 (0.0)	1 (6.7)	1.00
Intra-abdominal	1 (8.3)	1 (6.7)	1.00
Skin and soft tissue	3 (25.0)	6 (40.0)	0.68
Urinary tract	0 (0.0)	1 (6.7)	1.00
Mediastinitis	0 (0.0)	0 (0.0)	–
Bone and joint	2 (16.7)	1 (6.7)	0.57
Other and unknown	0 (0.0)	0 (0.0)	–

Metastatic infection, n (%)	4 (33.3)	4 (26.7)	1.00
Polymicrobial bacteremia, n (%)	1 (8.3)	3 (20.0)	0.60
Comorbid medical conditions, n (%)			
Malignancy	6 (50.0)	5 (33.3)	0.45
Diabetes mellitus	4 (33.3)	3 (20.0)	0.66
Chronic heart failure	2 (16.7)	2 (13.3)	1.00
Valvular heart disease	0 (0.0)	1 (6.7)	1.00
Chronic renal failure	5 (41.7)	1 (6.7)	0.06
Hemodialysis	2 (16.7)	2 (13.3)	1.00
Cirrhosis	0 (0.0)	0 (0.0)	–
Chronic pulmonary disease	0 (0.0)	3 (20.0)	0.23
Cerebrovascular event	0 (0.0)	1 (6.7)	1.00
Burn injury	0 (0.0)	2 (13.3)	0.49
Foreign body[a]	1 (20.0)	4 (26.7)	0.34
Immunosuppression[b]	0 (0.0)	3 (20.0)	0.23
Charlson comorbidity index, median (IQR)	4 (2.3–5.8)	2 (1–4)	0.10
Severity of infection			
Septic shock, n (%)	2 (16.7)	3 (20.0)	1.00
Mechanical ventilation, n (%)	1 (8.3)	3 (20.0)	0.61
Pitt's bacteremia score at onset of bacteremia, median (IQR)	0 (0–2)	1 (0–3)	0.41
SOFA score at onset of bacteremia, median (IQR)	2 (0–4.8)	3 (0–5)	0.64

Quick SOFA score at onset of bacteremia, median (IQR)	0.5 (0–1.8)	1 (0–1)	1.00
Antimicrobial therapy, n (%)			
Inappropriate empirical therapy	11 (91.7)	12 (80.0)	0.61
Initial anti-MRSA therapy, n (%)			
Vancomycin	5 (41.7)	7 (46.7)	1.00
Teicoplanin	3 (25.0)	2 (13.3)	0.63
Arbekacin	0 (0.0)	0 (0.0)	–
Linezolid	2 (16.7)	4 (26.7)	0.66
Daptomycin	2 (16.7)	1 (6.7)	0.57
Management, n (%)			
Removal of intravascular device within 5 days after the onset of bacteremia in the patients with CRBSI	7/7 (100.0)	7/8 (87.5)	1.00
Follow-up blood culture	12 (100.0)	15 (100.0)	–
Anti-MRSA therapy duration ≥14 days	11/12 (91.7)	13/15 (86.7)	1.00
Anti-MRSA therapy duration, median (IQR)	24.5 (15–46)	24 (16–33)	0.92
Outcome, n (%)			
Persistent bacteremia	1 (8.3)	6 (40.0)	0.09
All-cause 30-day mortality	1 (8.3)	1 (6.7)	1.00
All-cause in-hospital mortality	2 (16.7)	3 (20.0)	1.00

[a] Including prosthetic valve, vascular graft, joint, or pacemaker.

[b] Consisted of transplantation, AIDS, and use of corticosteroids or nontransplant immunosuppressive medications.

CA-MRSA, community-associated methicillin-resistant *S. aureus*; IQR, interquartile range; BMI, body mass index; CRBSI, catheter-related bloodstream infection; SOFA, Sequential Organ Failure Assessment; MRSA, methicillin-resistant *S. aureus*.
